# A Post-Processing Method for Three-Dimensional Electrical Impedance Tomography

**DOI:** 10.1038/s41598-017-07727-2

**Published:** 2017-08-03

**Authors:** Sébastien Martin, Charles T. M. Choi

**Affiliations:** 1 Department of Electrical and Computer Engineering, National Chiao Tung University, Hsinchu City, 30010 Taiwan; 2 Institute of Biomedical Engineering, National Chiao Tung University, Hsinchu City, 30010 Taiwan

## Abstract

Electrical impedance tomography is a modern biomedical imaging method. Its goal is to image the electrical properties of human tissues. This approach is safe for the patient’s health, is non-invasive and has no known hazards. However, the approach suffers from low accuracy. Linear inverse solvers are commonly used in medical applications, as they are strongly robust to noise. However, linear methods can give only an approximation of the solution that corresponds to a linear perturbation from an initial estimate. This paper proposes a novel reconstruction process. After applying a linear solver, the conductivity distribution is post-processed with a nonlinear algorithm, with the aim of reproducing the abrupt change in conductivity at the boundaries between tissues or organs. The results are used to compare the proposed method with three other widely used methods. The proposed method offers higher quality images and a higher robustness to noise, and significantly reduces the error associated with image reconstruction.

## Introduction

Electrical impedance tomography (EIT) is an imaging technique mostly used in biomedical imaging^1^. It involves estimating the electrical impedance within a volume conductor by injecting an electrical current at the boundary of the volume and measuring the resulting potential there^2^. Different living tissues have different electrical properties, and therefore EIT is applicable for biomedical imaging. Although first mentioned in the 1970s^3^, in practice, EIT is a relatively new technology, mostly because the high quality, inexpensive hardware, powerful algorithms and computational resources necessary to solve the problem are only emerging^4^.

An EIT inverse problem is highly nonlinear and very ill-posed^5^, and therefore the solution is not trivial and usually requires a certain conductivity distribution^6^ as an initial estimate of the solution. Several methods have been proposed to solve this inverse problem^7^, each having advantages and disadvantages^8^. In short, those methods can be regrouped in different classes: linear approximation^9^, nonlinear iterative methods^10^, direct nonlinear methods^11^ and machine learning (ML)-based methods^12^. With the linear methods, the conductivity distribution is approximated as a small perturbation from an initial estimate^13^. By assuming an initial conductivity distribution, such as by using a regularisation method or prior distribution, a satisfactory solution to the inverse problem can be better approximated by the reconstruction algorithm. Nonlinear algorithms are theoretically capable of higher accuracy and do not extensively rely on an initial guess, as linear methods do. However, nonlinear methods are more sensitive to the electrode displacements, modelling errors and time-varying contours of the imaged region, making them less reliable in most biomedical applications^14^. Although prior probability functions^15^ can be used to perform more accurate reconstructions in the presence of noisy data, for these methods, the inverse problem remains ill-posed and the presence of minor modelling errors, even in a very small amount, may lead to large artefacts in the image^16^.

Artificial neural networks (ANNs) are artificial intelligence (AI) algorithms capable of finding a better approximation of the solution to a nonlinear problem^17^. Applications of EIT inverse problems have shown the ability of ANNs to solve the inverse problem in a very short time^18^. However, as ANNs are based on ML, it appears complicated to generate correct training data for real biomedical applications^10^. The main weakness of ANNs is their inability to extrapolate and estimate solutions from previously unseen data. The latter is the major drawback of using an ANN to solve the EIT inverse problem in a biomedical environment. In real clinical data, the amounts of noise and modelling errors are not negligible, and therefore training an algorithm to model the conductivity distribution is infeasible, as the amount of noise cannot always be evaluated a priori.

**Figure 1:**
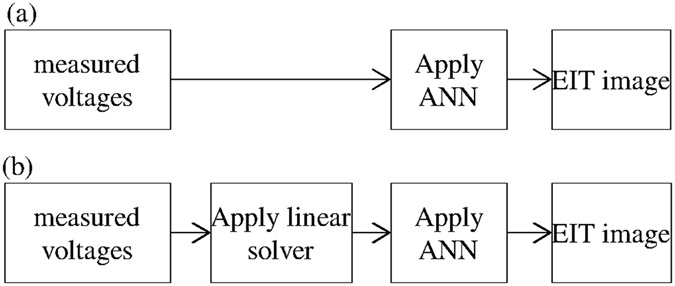
EIT Comparison between (**a**) existing reconstruction methods using an ANN and (**b**) the proposed method.

This paper presents a novel approach to solving a three-dimensional EIT problem. This approach mixes both linear approximations and an ANN in the inverse problem, and aims to combine the benefits of both linear approximations and nonlinear optimisations. After solving the inverse problem with the linear one-step Gauss-Newton (GN) algorithm^19^, a linear distribution is obtained. The idea is to use an ANN as a post-processing method to overcome the weaknesses of the inverse solver and rectify the conductivity distribution. Applying the ANN after solving the EIT inverse problem should confer a significantly higher robustness to noise. Although applying an ANN directly on the measured voltages requires the correct modelling of the noise and the possible distortions that appear in the measured voltages, applying it to the output of a linear reconstruction algorithm does not require such an intense modelling task to provide a satisfactory image. In fact, the linear algorithms are known to be extremely robust to noise, and therefore the resulting conductivity distribution is not strongly influenced by the modelling errors compared with the measured voltages. Therefore, training an ANN to enhance the quality of the reconstruction as a post-processor, rather than using an ANN as an image reconstruction processor, should provide a stronger robustness to measurement errors present in the measured data, even when they are not accounted for during the training phase. A comparison between existing methods based on the ANN and proposed method is shown in Fig. 1.

The proposed method is compared with three widely used methods: a linear method^19^, a nonlinear iterative method^20^ and an inverse solver based on an ANN^21^. The results show that the proposed method is efficient, stable and rapid.

## Results

### Phantom experiments

**Figure 2:**
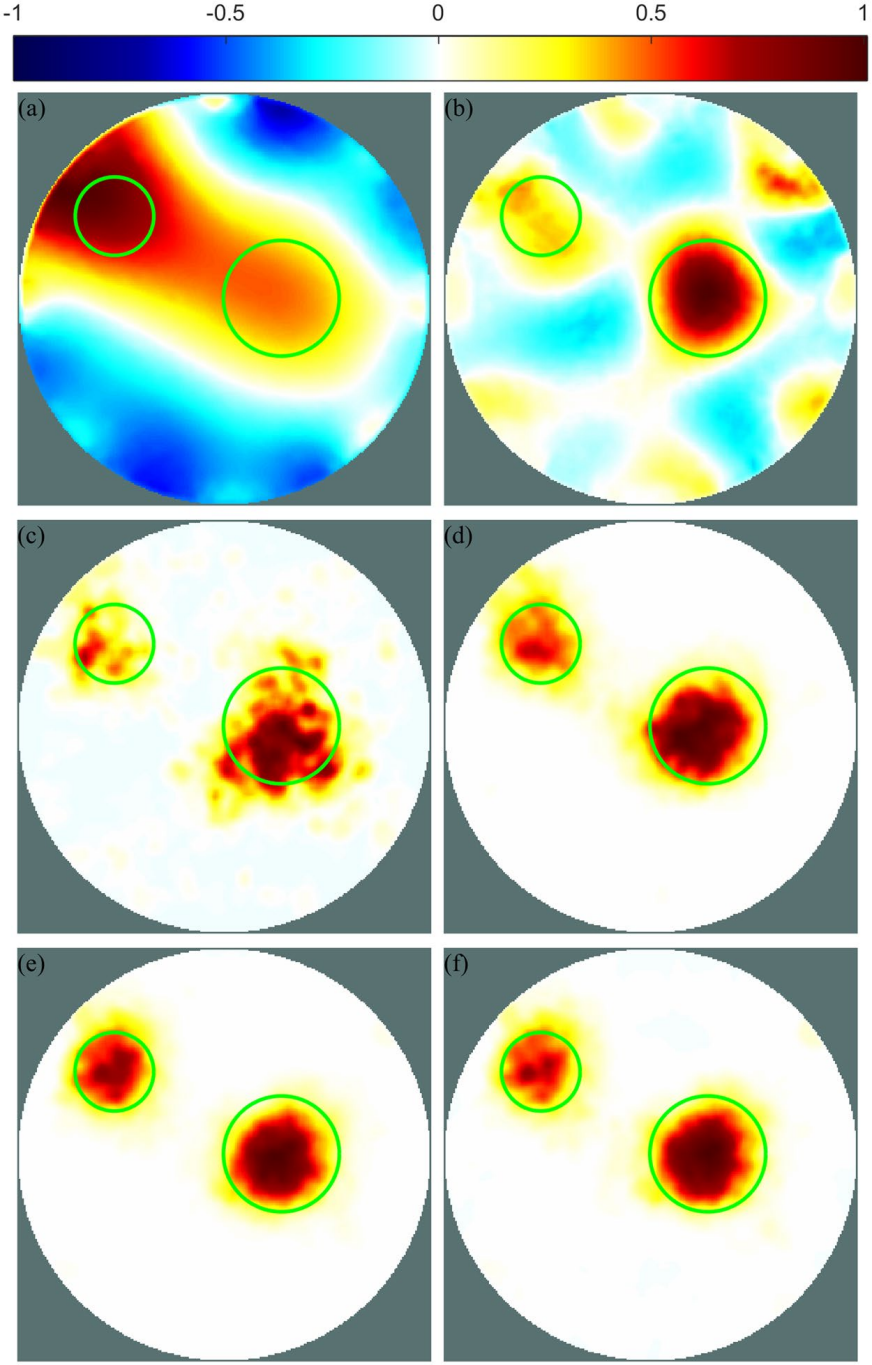
Cross-section view of EIT reconstructions from phantom data with different methods: (**a**) the one-step GN, (**b**) the PDIPM, (**c**) an ANN as inverse solver trained without considering noise, (**d**) the proposed post-processing method and an ANN trained without considering noise, (**e**) an ANN as inverse solver trained with noisy data and (**f**) the proposed post-processing method and an ANN trained with noisy data. Green circles show the location of the targets. The bar at top is the normalised conductivity distribution.

Phantom experiments were carried out with a cylindrical tank filled with ionised water. Two electrical insulators made of acrylic were inserted into the phantom and EIT measurement data were collected. EIT image reconstruction was then performed with four different methods: the linear one-step GN, the iterative primal dual interior point method (PDIPM) solver, an ANN used as an inverse solver and the proposed post-processing method. Cross-sections of the resulting images are shown in Fig. 2 and the 3D models are shown in Extended Data Fig. 1. The linear one-step GN method correctly outputs two different targets near the expected location, but close to the boundary of the finite element (FE) model. The linear algorithm generates smoothness in the conductivity distribution, which makes it difficult to correctly represent the two targets without generating large artefacts. Here, the smoothness present in the underlying algorithm tends to generate large ringing artefacts, which are responsible for the presence of the blue colour in the reconstruction. These artefacts are very likely to lead to an incorrect interpretation of the conductivity distribution.

The iterative PDIPM method shown in Fig. 2(b) correctly shows two different targets at the expected location, represented by the green cylinders. The use of a nonlinear algorithm does not give a smooth conductivity distribution and therefore the ringing artefacts, shown by the blue objects on the image, are significantly reduced compared with the linear one-step GN. An ANN is applied to solve the EIT inverse problem in Fig. 2(c) and (e). If the ANN is trained without considering the inevitable presence of noise in the measured data, image reconstruction from noisy data may generate large errors. Figure 2(c) shows the reconstruction obtained from phantom data with such an ANN. Although the ANN does not generate visible ringing errors, it does not clearly show the two expected cylindrical objects, but gives distorted objects. This poor image reconstruction was expected from the theory as well as from other studies, which have shown that ANNs are capable of performing a satisfactory reconstruction and also very sensitive to modelling errors and noise present in the measured data. In other words, when the training data do not include noise similar to the noise present in measurement data, the ANN must extrapolate the result and therefore gives a poor reconstruction. In Fig. 2(e), the ANN is trained by considering the presence of noise in the measured data. The conductivity distribution obtained with this ANN gives a satisfactory reconstruction. The two different objects are represented as two electrical insulators, and their estimated positions seem to match the initial position. Furthermore, neither visible distortion nor ringing artefacts are present.

The proposed method, which combines both the linear reconstruction method and ANN, was tested on the same measurement data. In the proposed method, the ANN was trained with EIT reconstructions from the one-step GN solver, obtained with simulated data. The image in Fig. 2(d) was obtained using the proposed post-processing method and an ANN trained without considering the presence of noise in the measured data. Compared with an ANN used as an inverse solver trained without considering the presence of noise in the measurement data, as in Fig. 2(c), the proposed method performed better. When solving the inverse problem with a linear algorithm, such as the one-step GN, the imperfectly modelled and constantly changing electrodes position, contact impedance and model contours^22^ had a limited effect on the image. Thus, for the proposed post-processing method, training the ANN without considering the presence of noise in the measured data still led to a satisfactory image, close to the image obtained with an ANN trained by considering noisy measurement data.

For the last reconstruction, shown in Fig. 2(f), noise was added to the simulated voltage data so that these voltages would be similar to the voltages measured from the phantom and therefore the need for extrapolation by the ANN would be reduced. Compared with the proposed post-processing method and an ANN trained from noise-free data, this solution gave a similar image. The two targets are visible at the expected location. The smoothness and the ringing effects or shape deformation are not visible to the human eye. As for the ANN used as an inverse solver trained from noisy voltages, the ANN applied as a post-processing method resulted in a satisfactory reconstruction without visible smoothness or ringing artefacts. In addition, the resulting images show that the two reconstructed objects did not shift towards the boundary of the FE model, as may happen with linear methods.

For each reconstruction, different errors^23^ were calculated to validate the visual impression that the proposed post-processing with ANN offered a strong resistance to the noise present in the measurements. The corresponding errors are given in Table 1.

**Table 1:** PE, |ΔRES| and SD errors obtained for reconstruction from phantom data with different methods.

Method	PE #1 (%)	PE #2 (%)	|ΔRES| (%)	SD (%)
One-step GN	2.69	2.30	19.44	20.03
PDIPM	1.95	2.10	8.10	22.91
ANN (training: no noise)	2.60	1.76	6.90	17.87
One-step GN + ANN (training: no noise)	1.35	0.55	2.59	11.21
ANN (training: noise)	0.97	0.37	2.35	10.35
One-step GN + ANN (training: noise)	1.01	0.50	2.27	10.55

Corresponding images are shown in Fig. 2. Target #1 is located on the left side and target #2 is located on the right side of the images.

Position error (PE) indicates an error on the position of the target. PE was divided into two errors, one for each different target. On the images shown in Fig. 2, the target on the left is called the first target, and the target on the right is called the second target. PE errors are normalised based on the radius of the FE model. For the first target, the one-step GN method tended to push the reconstruction towards the boundary of the FE model, which increased the PE and was as high as 2.69% in this case. The ANN used as an inverse solver trained without considering the presence of noise also gave a poor result, as seen in Fig. 2(c), with a PE of 2.60%. Other methods were able to estimate the position of the target with high accuracy, and the PE obtained from ANN-based methods did not exceed 1.35%. Similar to the first target, the linear one-step GN and the ANN used as an inverse solver trained without noisy data gave the two highest PE for the second target. The one-step GN gave an error of 2.30%, while the ANN used as an inverse solver trainer gave an error of 1.76% when trained without considering noise. The ANN used as an inverse solver trained with noisy data gave the best estimate, with an error of 0.37%. Post-processing methods did not exceed 0.55% (obtained with training without noise), less than 0.2% more than the ANN used as an inverse solver trained with noisy data.

Difference of resolution (|ΔRES|) errors can be interpreted as the difference between the area of the target and the area of the reconstructed object. The one-step GN method generated large smoothness, which leads to a large |ΔRES| of 19.44%. The proposed method gave the lowest error of 2.27% if trained by considering the presence of noise. When trained correctly, an ANN used as an inverse solver also gave a low error of 2.35%. However, if the ANN was not trained correctly, the error increased to 6.90%. The proposed post-processing gave a low error in both cases, regardless of whether the presence of noise was (2.27%) or was not (2.59%) considered during training. Additional |ΔRES| errors, obtained at different heights of the 3D model, are presented in Extended Data Table 1, and show that the proposed method performed better than other methods at different cross-sections, as can be seen from Extended Data Fig. 1.

Similarly, the linear one-step GN method and PDIPM both gave large shape deformation (SD) errors of 20.03% and 22.91%, respectively. An ANN used as an inverse solver gave a very low error of 10.35% if trained while considering the presence of noise in the measurements. If not, the SD error could go as high as 17.87%. Although training the ANN with noisy voltages always gave a slightly better result, the proposed post-processing method also gave a low error when trained without any consideration of the noise. In this case, the SD varied by less than 1%, from 10.55% when trained with noisy data to 11.21% when trained with non-noisy data. This observation shows the high stability and large resistance to noise of the proposed method.

To summarise, Table 1 shows that the proposed method is capable of giving an accurate reconstruction of the EIT inverse problem without requiring a strong a priori knowledge of the data acquisition system.

Regarding the cost of computation, the one-step GN solver consists of a simple matrix product. Computing the matrix requires 48.6 GB of memory and takes 3,029.18 seconds. However, the matrix does not depend on the measurements and can therefore be calculated before solving the inverse problem. In this example, the reconstruction matrix was considered as known when applying the one-step GN solver.

The time and memory required to solve the EIT inverse problem by different methods are given in Table 2. Once the reconstruction matrix was known, the EIT inverse problem could be solved within 0.1 s, and with only 0.6 GB using the one-step GN method. The PDIPM method is an iterative approach and requires both time and a large amount of memory to achieve a reconstruction. This solver converges to a precise reconstruction, but takes 4,289 seconds and 65.46 GB of memory to solve the problem. Here, the method based on ANN as an inverse solver was the fastest and only took 0.36 s and 0.38 GB. The proposed method combines the one-step GN solver and an ANN, and therefore takes longer than these two solvers. Here, the proposed method solved the inverse problem in 0.80 s and needed only 1.1 GB of memory.

**Table 2:** CPU time and memory required to solve the EIT inverse problem from phantom data with different methods.

Method	CPU Time (s)	Memory (GB)
One-step GN	0.09	0.59
PDIPM	4289.57	65.46
ANN as inverse solver	0.36	0.38
One-step GN + ANN	0.80	1.10

Although the PDIPM is also capable of solving the EIT problem with great accuracy, its iterative approach requires both time and resources, and therefore this solution is not applicable for fast imaging applications. Compared with the proposed post-processing method, the iterative PDIPM approach is more than 4,000 times slower and takes more than 100 times the amount of memory needed to solve the inverse problem. Therefore, expensive hardware must be used, which is a significant drawback.

### Lung data

**Figure 3:**
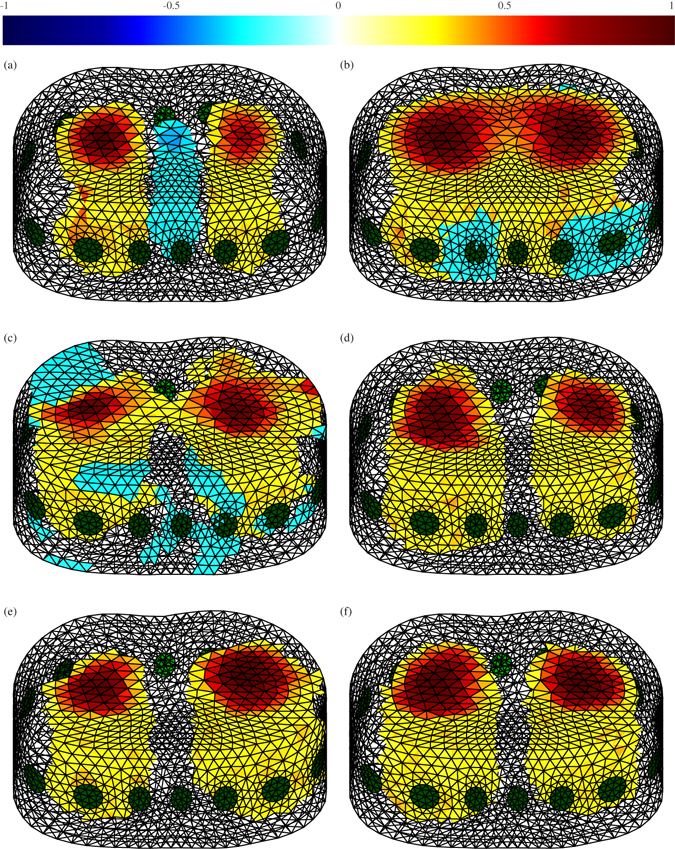
3D EIT reconstructions of lung data using different methods: (**a**) one-step GN, (**b**) the PDIPM, (**c**) an ANN as inverse solver, trained by considering sources of errors, (**d**) the proposed method, trained by considering errors in measurement data, (**e**) an ANN as inverse solver, trained without considering errors and (**f**) the proposed post-processing method, trained without considering errors. The normalised resistivity distribution is given at the top. The electrodes are shown in green.

EIT data of a healthy person were collected^24^ and the images reconstructed using the same four methods. In this experiment, 16 electrodes were located around the subject’s chest in a single plane. As the subject was healthy, two similar elliptical shapes were expected to represent the lungs. Figure 3 shows the conductivity distributions within the FE models obtained from lung data using the different methods. Extended Data Fig. 2 shows a cross-section from these FE models. Extended Data Fig. 2 shows the conductivity distribution at the middle of the FE model, where the electrodes were located.

The one-step GN method, widely used in real-time biomedical applications, gave an image showing two different targets; however, it also contained smoothness and therefore some artefacts that degraded the quality of the reconstructed image. Ringing artefacts were visible between the two lungs. These artefacts, represented by the blue region visible in Fig. 3(a), could have led to an incorrect interpretation of the result and a wrong diagnosis by the practitioner and thus had to be eliminated.

The PDIPM result in Fig. 3(b) shows a fused lung, whereas the plots in Fig. 3(c–f) show separated lungs. Figure 3(c) is distorted and Fig. 3(e) is slightly distorted when compared with Fig. 3(a), which is the established standard. Moreover, Fig. 3(d) and (f) are much superior to Fig. 3(a) and the rest of the plots in Fig. 3.

When using an ANN as an inverse solver, the result strongly depended on the simulated data used to train the ANN. Basically, the closer they were to the measured data, the better the result. Based on this statement, training an ANN to give excellent EIT image reconstruction appeared to require an intense modelling effort. As this study considered time difference EIT, the modelling had to consider not only the breathing activity of the patients, but also the presence of movement and model distortion through time, which led to electrode displacement. In addition, research has shown that, to give a better result, modelling work should consider the presence of noise in the measured data, which is hardware-dependent. Figure 3(c) and (e) both show the reconstruction with an ANN used as the inverse solver. In Fig. 3(c) the ANN is trained by omitting the presence of noise, electrode movement and model deformations, while in Fig. 3(e) the ANN is trained by considering the presence of those artefacts. Data were collected at the end of the inspiration and expiration phases, meaning that two similar targets representing the conductivity difference during the breathing cycle should be visible on the image. If the ANN is trained while considering the different sources of errors, as in Fig. 3(e), two different elliptical regions are visible. Although the left lung appears to be slightly smaller than the right, their sizes are relatively close and they can be separated easily. However, when the ANN is trained without considering the errors and inaccuracies of the hardware, as in Fig. 3(c), large artefacts may appear, shapes are not consistent with expectations and it becomes difficult to correctly separate the two different regions.

As for the phantom experiments, the proposed method was less sensitive to the presence of noise, movement and measurement errors. Figure 3(d) and (f) show images obtained with the proposed method by not considering the different sources of errors during the training phase and considering them, respectively. In both cases, the two lungs are visible and separated. Some artefacts are visible in Fig. 3(d), where the ANN is trained without considering any source of error in measurement, but the visual quality of the reconstruction is significantly improved compared with the reconstruction based on the ANN only, as shown in Fig. 3(c). Compared with other the nonlinear methods used in this paper, the proposed method gave significantly fewer artefacts. Finally, Fig. 3(f) gives the best image of the conductivity difference in the lungs during the breathing cycle; two large symmetrical shapes can be seen, the smoothness remains low and there is no visible artefact on the image. However, even when considering modelling errors during the training of the ANN improved the result, this step was not necessary to obtain a satisfactory image of two separate targets similar to the lungs. This finding contributes to considerably reducing the modelling effort required to train an ANN efficiently for biomedical applications.

For each method, the computational resources used to perform 3D EIT reconstruction were measured. Although the iterative PDIPM method generally offers high quality, it takes a long time and cannot be used for real-time monitoring. Compared with the PDIPM method, the one-step GN- and ANN-based methods are significantly faster. The proposed method is capable of solving the inverse problem in less than 0.3 second on a CPU and is expected to be even faster on a GPU or specific hardware. CPU time and memory consumption are given in Table 3.

**Table 3:** CPU time and memory required to solve the EIT inverse problem from lung data with different methods.

Method	CPU Time (s)	Memory (MB)
One-step GN	0.03	70
PDIPM	5.04	669
ANN as inverse solver	0.13	236
One-step GN + ANN	0.29	413

To show the stability of the proposed method, different measurements were performed during the breathing cycle and each individual image was reconstructed. The results are recorded in Extended Data Figs 3–6. Although the one-step GN method was stable, the images show relatively small lungs and ringing artefacts between them. The PDIPM method gives high quality images but also generates large artefacts instead of blank images when both the reference and measured signals are acquired during the same phase of the breathing cycle (i.e., the end of expiration). The ANN used as an inverse solver appeared to be stable but generated strange shapes, different from the expected shape of the lungs. Finally, the proposed method offered a stable and accurate reconstruction. The resulting images show a complete breathing cycle with two lungs having similar sizes and a satisfactory shape close to the shape obtained from other imaging modalities such as computerised tomography.

## Conclusion

In this paper, a novel reconstruction method for 3D EIT is proposed. By offering near real-time image reconstruction, strong robustness against noise and rough boundaries, the proposed method combines the advantages of both linear and nonlinear methods. Although simulation data do not show significant amelioration between the existing methods based on the ANN and novel method presented in this paper, phantom and lung data clearly show the advantages of the new method, especially its ability to produce high quality images from a noisy environment. Solving the EIT problem with linear solvers before applying the ANN helps to reduce the influence of noise present in the measured data and confers higher stability to the nonlinear ANN. This higher stability then allows the training of an ANN to solve the EIT inverse problem from biomedical data without an extensive knowledge of either the physiology of the human anatomy or the hardware used for EIT data acquisition. Compared with a solution based on an ANN only, the proposed method provides significantly greater stability and stronger robustness to previously unseen data. The results show that, with the proposed method, a deep knowledge of the data acquisition system, as well as the patient’s body shape, are no longer necessary to train an ANN capable of giving a high resolution image. Based on this observation, the computationally expensive training process can be done only once, and then an identical set of weights and biases can be used for every patient and EIT hardware available. In this study, the inverse problems were solved using Matlab and calculations were performed on a CPU. The authors believe that future computers and a GPU-based implementation of the proposed method should open the way to real-time implementation of a high quality, stable and non-linear EIT image reconstruction of the lungs.

## Methods

Phantom data were acquired from a physical phantom. The cylindrical tank contained four layers of electrodes. Each layer contained eight different electrodes, giving a total of 32 electrodes. Both current injection and measurements were made using an adjacent current pattern, meaning that each pair of electrodes used corresponded to two adjacent electrodes, located on the same layer. Phantom data were acquired using the data acquisition system introduced by Tu^25^. This system uses each pair of two adjacent electrodes located on the same layer for current injection. For each current injection, 32 different voltages were measured with the 32 pairs of adjacent electrodes present at the boundary of the volume conductor. The current source then moved to the next pair of adjacent electrodes, and 29 other measurements were acquired. Finally, 928 measurements were used for image reconstruction.

The difficulty presented by real-life EIT applications is to correctly model the contour of the volume conductor and the positioning of the electrodes. The real boundary shape not only is slightly different for every patient, but also changes continuously during breathing and therefore during the monitoring process. Due to the presence of errors in estimating the contour of the thorax region, additional errors result from the positioning of the electrodes. Additionally, due to the presence of movement during data acquisition, between the measurement of the reference signal and the actual frame, a reconstruction in a fixed FE model cannot give an exact solution, only approximate a solution. Nonlinear iterative algorithms are known to be highly sensitive to the mismatch between the boundary of the FE model and actual shape of the volume conductor.

A difference EIT approach involves using two different sets of measurements, usually acquired at two different times, and considering the difference between these two measurements to do the reconstruction. This approach helps to cancel out errors due to imperfect modelling. However, the inverse problem requires only one FE model to image the conductivity distribution. In other words, if the shape of the volume conductor keeps changing during measurement, the difference EIT cannot cancel out all of the errors resulting from imprecise modelling, only help to reduce them.

It has been shown that ANNs are capable of dealing with these issues when trained accordingly. To train an ANN capable of considering a possible modelling error usually present in practical biomedical applications, training data should include such possible artefacts. In this study, to train the ANNs from simulation data, the forward problems had to be solved with different FE models. For EIT imaging of the lung data, a circular FE model was generated and distorted using Fourier coefficients of the average human thorax. During the training phase, the Fourier coefficients were modified with a random weight of up to 10% of the original coefficient to obtain different shapes similar to the thorax. In addition, given the focus of this work on difference EIT, two different FE models were used to solve the forward problems in the homogeneous and inhomogeneous cases, respectively. The forward problems were then solved using different models. Finally, for the proposed post-processing solution, the inverse problem was solved in an FE model of the lungs obtained without modifying the Fourier coefficients.

The proposed method was compared with a reconstruction method based on an ANN only and two widely accepted methods for EIT reconstructions of the lungs.

### EIT inverse solvers

#### One-step gauss-newton

The one-step GN is a direct linear reconstruction method commonly used for real-time imaging applications^26^. This method offers the advantage of being non-iterative, and therefore a solution to the inverse problem can be computed in a very short time. This reconstruction method can be seen as a simplified linearised version of the nonlinear GN method, named after the mathematicians Carl Friedrich Gauss and Isaac Newton. In fact, only the first step of the nonlinear method is calculated. This solution gives a rapid and satisfactory reconstruction, as in difference EIT some parameters of the complete nonlinear method feature a very poor identifiability and therefore can be set to constant values.

#### Primal-dual interior point method

The nonlinear PDIPM is an iterative reconstruction method and therefore requires both time and large computing resources to estimate a solution to the inverse problem. The PDIPM is essentially based on barrier methods widely used in linear and nonlinear programming. The idea of encoding the feasible set using a barrier and designing barrier methods was studied by Anthony V. Fiacco, Garth P. McCormick and others in the early 1960s^27^. These methods fall into the category of simplex methods, in which the solution follows the boundary of the feasible set^28^. Karmarkar proposed a new algorithm called Karmarkar’s algorithm, which runs in provably polynomial time and is also very efficient in practice^29^. Compared with the simplex methods, this algorithm, later called the PDIPM, has the ability to search at the interior of the feasible set instead of at the boundary. Later, in 2012, Borsic and Adler proposed using the PDIPM method to solve the EIT inverse problem^20^ and obtained high quality image reconstructions, showing rough boundaries and nonlinear conductivity distributions. In this study, this algorithm was used with the L1 norm on the data and the L2 norm on the regularisation term.

Both the one-step GN solver and PDIPM solver were used with the well-known prior probability function initially used with the Newton one-step error reconstructor (NOSER) algorithm^30^.

### Noise estimate

To train the ANN, 2,000 EIT images containing random targets were simulated. For each of these conductivity distributions, both the forward and inverse problems were solved. Each of the 2,000 EIT images contained targets with random conductivity, different from the background. Solutions to the forward and inverse problems were obtained using the EIDORS toolkit^24^ and Matlab’s neural networks toolbox, running under an Intel Core I7-6700 CPU at 4 GHz with 64 GB of RAM and Ubuntu Linux. After solving the forward problem, noise was added into the simulated voltages. The amount of noise added was determined by analysing the measured signals at the electrodes.

Although most of the noise was eliminated using a tenth-order bandpass filter centred on the frequency of the injected current, some noise was still present in the acquired data. To reduce the need to extrapolate from the ANNs, it was of interest to train them with noisy data similar to the data acquired from the phantom.

The amount of noise was not fixed and strongly depended on the physical distance between the current source and the electrodes used for measurement. Noise was estimated independently for each of the phantom experiment and lung data measured. Current injection consisted of a sinusoidal waveform at a frequency of 100 kHz. During each sine wave, 20 voltages were measured by each pair of electrodes. For EIT reconstruction, these data were filtered and the highest peak was considered. The noise was then estimated by comparing the measured data with a simulated sine wave. By estimating the amount of noise in the measurements before filtering, the noise was assumed to be a WGN. Noise was estimated according to (1):

1 $$SNR\,(dB)=10\ast \,\mathrm{log}(\frac{mean(signa{l}^{2})}{mean(residual\,nois{e}^{2})})$$

Finally, the signal-to-noise ratios for each of the 928 measurements were estimated over 500 different frames and averaged. When the measured signal was spatially close to the injected current, the estimated SNR was above 50 dB. However, when it was measured at the opposite side of the phantom, the SNR could be less than 10 dB, basically a result of the attenuation of the medium. By assuming a WGN before filtering, it became possible to reproduce the noise by adding a WGN to the simulated sine waves. After that, simulating the presence of a filter generated a noise model that was close to the noise present in the real phantom experiments.

For the lung measurements, internal organs and movements could also be responsible for the presence of noise. Therefore, a non-Gaussian noise was considered. The Fourier transform of the measured signals were analysed and a non-Gaussian noise was added to the measurement data.

### Artificial Neural Networks

Radial basis functions (RBFs) were used in the hidden layer of neurons. The output layer was made of a linear transfer function. Research has shown that this configuration of an ANN is capable of high quality EIT reconstruction from biomedical data. For an ANN used as an inverse solver, the input layer has a number of neurons equivalent to the number of measurements (e.g., 208 neurons for the lung data, 928 neurons for the phantom data). For the post-processing application, the input layer has a number of neurons equivalent to the number of nodes present in the FE model. In this study, in both cases, the hidden layer was made of 1,000 neurons with a RBF transfer function. Finally, the output layer outputted the estimate of the conductivity distribution within the FE model and contained a number of neurons equivalent to the number of nodes present in this model.

To train the ANN for the phantom data, training data were simulated. In those simulated data, the presence of one or two cylindrical insulators within a saline solution was simulated. Each of the insulators had a random electrical conductivity, defined to be lower than the background. In those simulations, the electrical conductivity of the homogeneous background was not fixed and varied randomly across the different training samples. After simulating up to 2,000 different conductivity distributions, the forward problems were solved with and without considering the presence of distortions (movement). When solving the EIT forward problems, a second-order forward solver and a very fine FE model different from the model used to solve the inverse problems were used to avoid committing the inverse crime.

In the case of the lungs, each lung was considered to be an electrical insulator compared with the background conductivity. Research has estimated that the electrical conductivity of the lungs is higher at the end of the expiration phase than at the end of the inspiration phase. According to previous studies, at the end of expiration, the lungs are expected to have an electrical impedance of approximately 700 Ω.m at the frequency of the injected current, or 100 kHz. At the end of the inspiration phase, the electrical impedance is expected to rise as high as 2,500 Ω.m. The electrical properties of the other tissues and materials crossed by the electrical current may also be influenced by several factors such as the heartbeat or blood circulation within the body. The difference in conductivity between the two different current injections (when a different pair of electrodes is used for measurement) is expected to remain low and is therefore neglected during the training of the ANN. In this study, the background impedance was arbitrarily set to be 700 Ω.m. As difference EIT was used in this application, the difference of conductivity in the background should not have been visible. In other words, the absence of change in the conductivity around the spinal cord and blood vessels between two different measurements made them invisible in the reconstructed image. Therefore, such artefacts were neglected during the generation of the training samples. Although in real biomedical applications the presence of movements and variation of blood pressure may theoretically be reflected in the final image, neglecting them allows for great simplification in the generation of 2,000 training samples. The RBF ANNs were trained by simulating the presence of two elliptical cylinders of random size and conductivity varying within the range of the possible electrical conductivities determined in previous work. After training with the particle swarm optimisation method, the resulting ANN assumed the presence of two lungs, a seemingly reasonable assumption for biomedical imaging. The forward and inverse problems were solved using widely accepted methods for biomedical EIT applications.

### Errors

Different functions have been proposed for use as an error function for medical imaging applications^31–33^, and a consensus (Graz consensus reconstruction algorithm for EIT, or GREIT) aimed to propose normalised error definitions^23^. Among these normalised definitions, the position error (PE), the resolution (RES) error and shape deformation (SD) were of significant interest and were calculated. These normalised definitions were designed for 2D EIT but can easily be adapted to a 3D EIT problem. In 2D EIT, these errors are estimated based on a rasterised image of the FE model. In this study, the resulting errors were measured on different cross-sections of the FE model. As RES was expected to give an estimate of the area of the target, the difference of resolution |ΔRES|^18^ was used.

If more than one target was present in the phantom, a PE was computed for each individual target, based on the method described by Martin and Choi^21^.

## Electronic supplementary material

Supplementary Information
