# Supplementary material for: A Post-Processing Method for Three-Dimensional Electrical Impedance Tomography
Source: Sci Rep. 2017 Aug 3;7:7212. doi: 10.1038/s41598-017-07727-2 (PMC5543057; doi:10.1038/s41598-017-07727-2)
Supplement: Supplementary file 1 — Supplementary Information [file 41598_2017_7727_MOESM1_ESM.pdf]

A Post-Processing Method for Three Dimensional Electrical Impedance Tomography

Sébastien Martin and Charles T.M. Choi

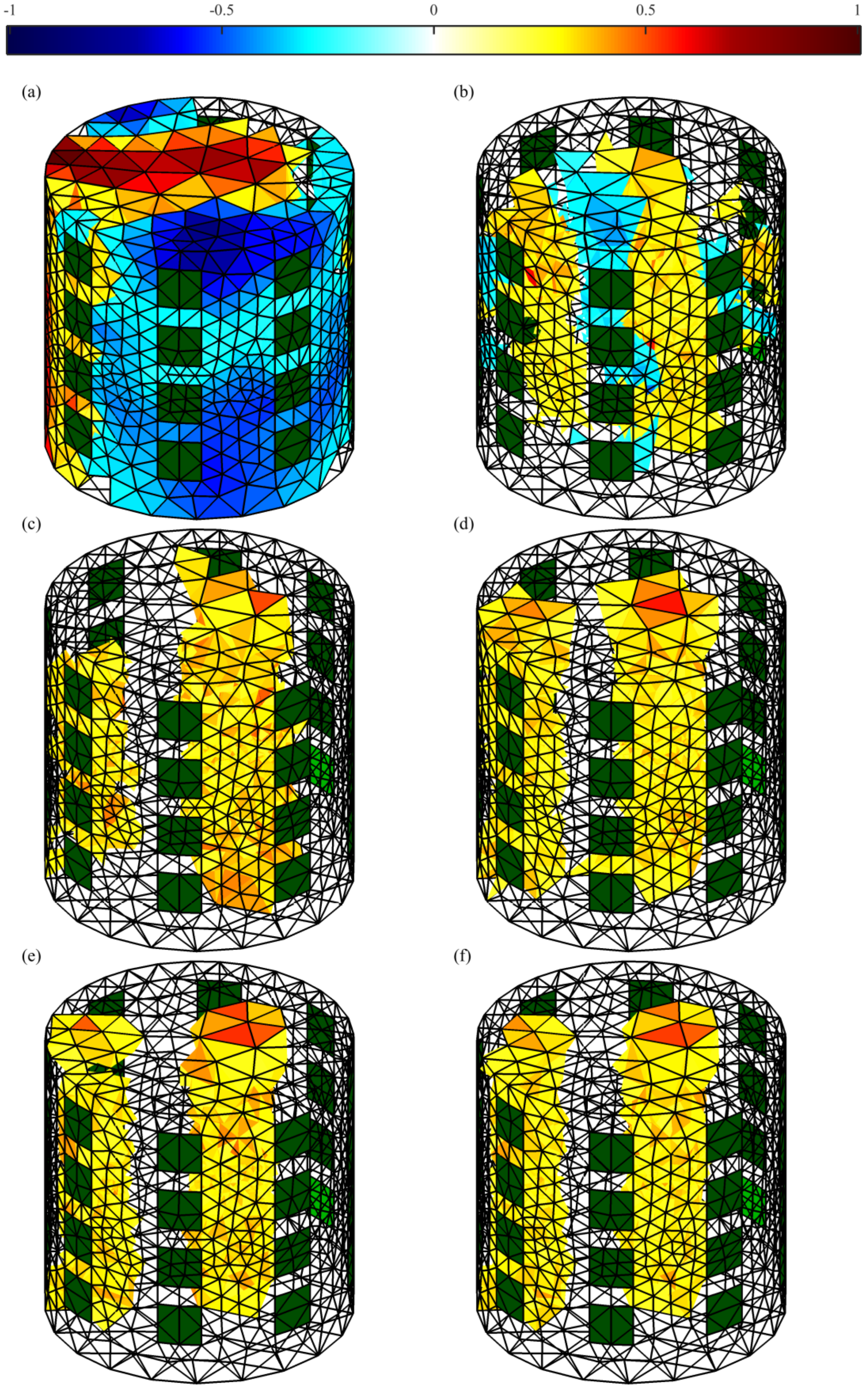

Extended Data Figure 1 EIT image reconstructions from phantom experiments with different algorithms: (a) the one-step GN, (b) PDIPM, (c) ANN as inverse solver trained without noisy data, (d) proposed post-processing method and ANN trained without noisy data, (e) ANN as inverse solver trained to consider noise, and (f) proposed post-processing and ANN trained to consider noise. The two targets present in the phantom are shown in (a). On top, the normalized resistivity distribution.

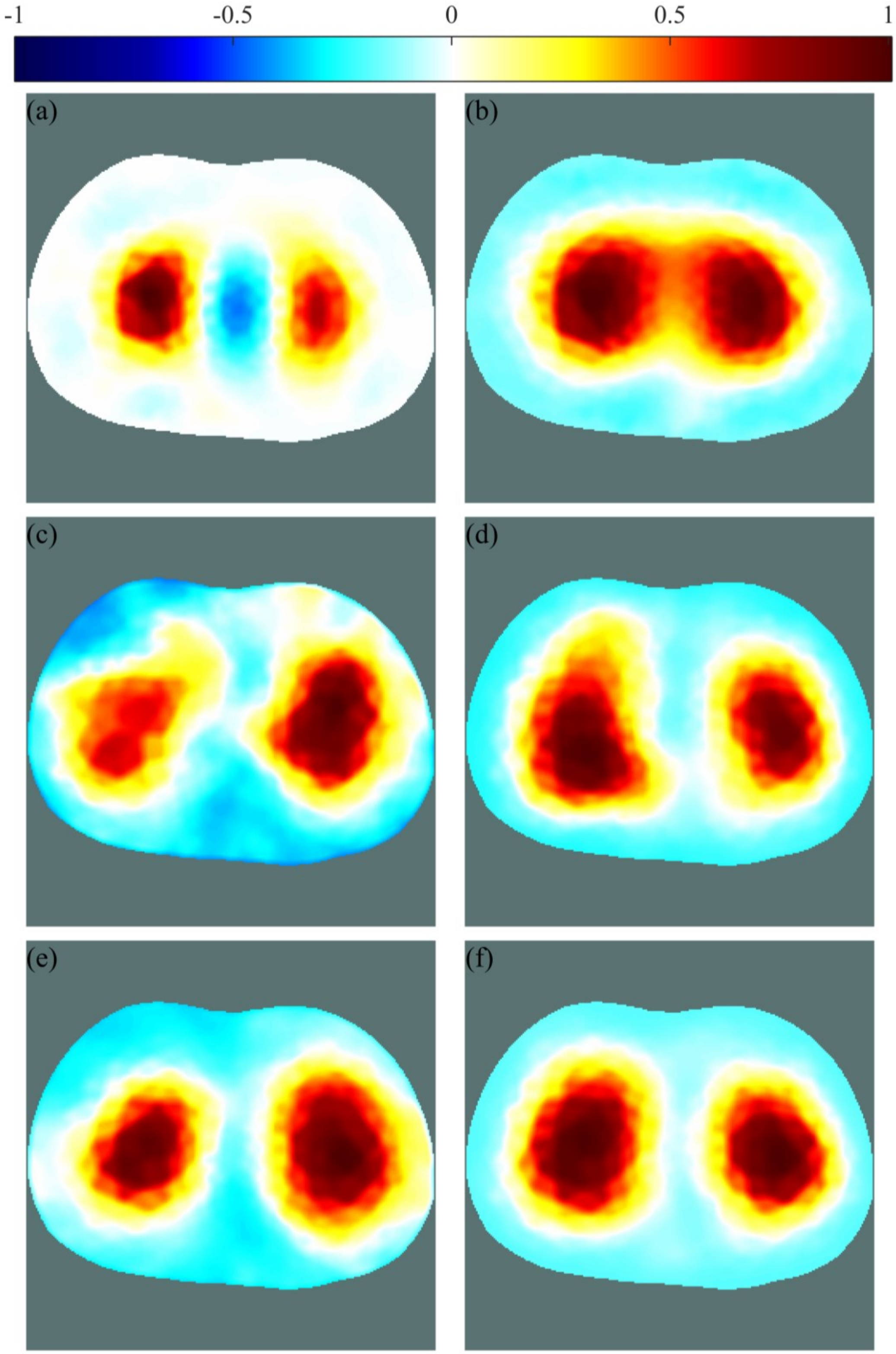

Extended Data Figure 2 Cross-section from FE models presented in Figure 3. Reconstructions were obtained with; (a) one-step GN, (b) PDIPM, (c) ANN as inverse solver, trained without considering sources of errors, (d) the proposed method, trained without considering errors in measurement data, (e) ANN as inverse solver, trained by considering errors and, (f) the proposed post-processing, trained by considering errors. Bar at the top is the normalized resistivity distribution.

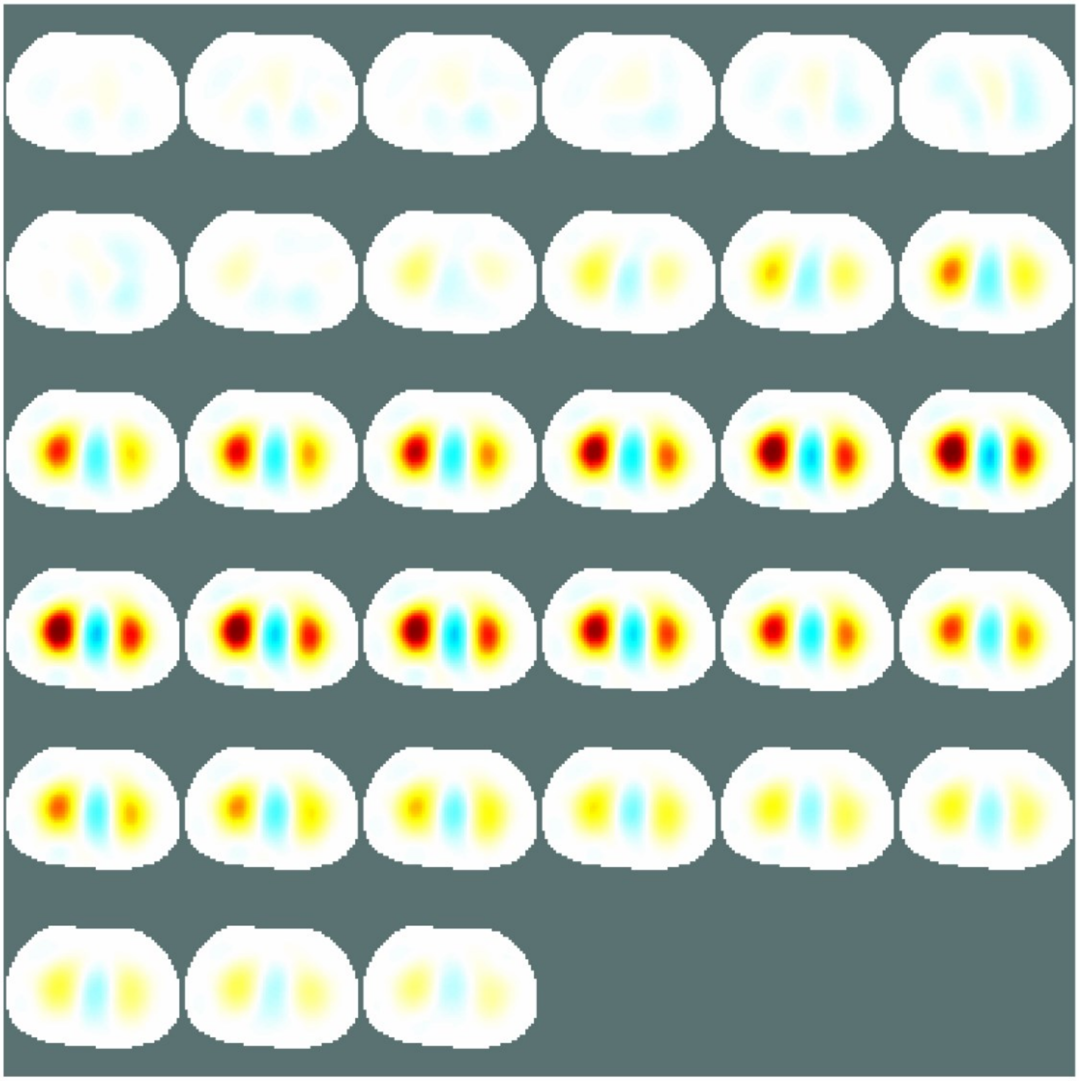

Extended Data Figure 3 33 EIT reconstructions with the one-step GN method, representing a complete breathing cycle from a healthy patient

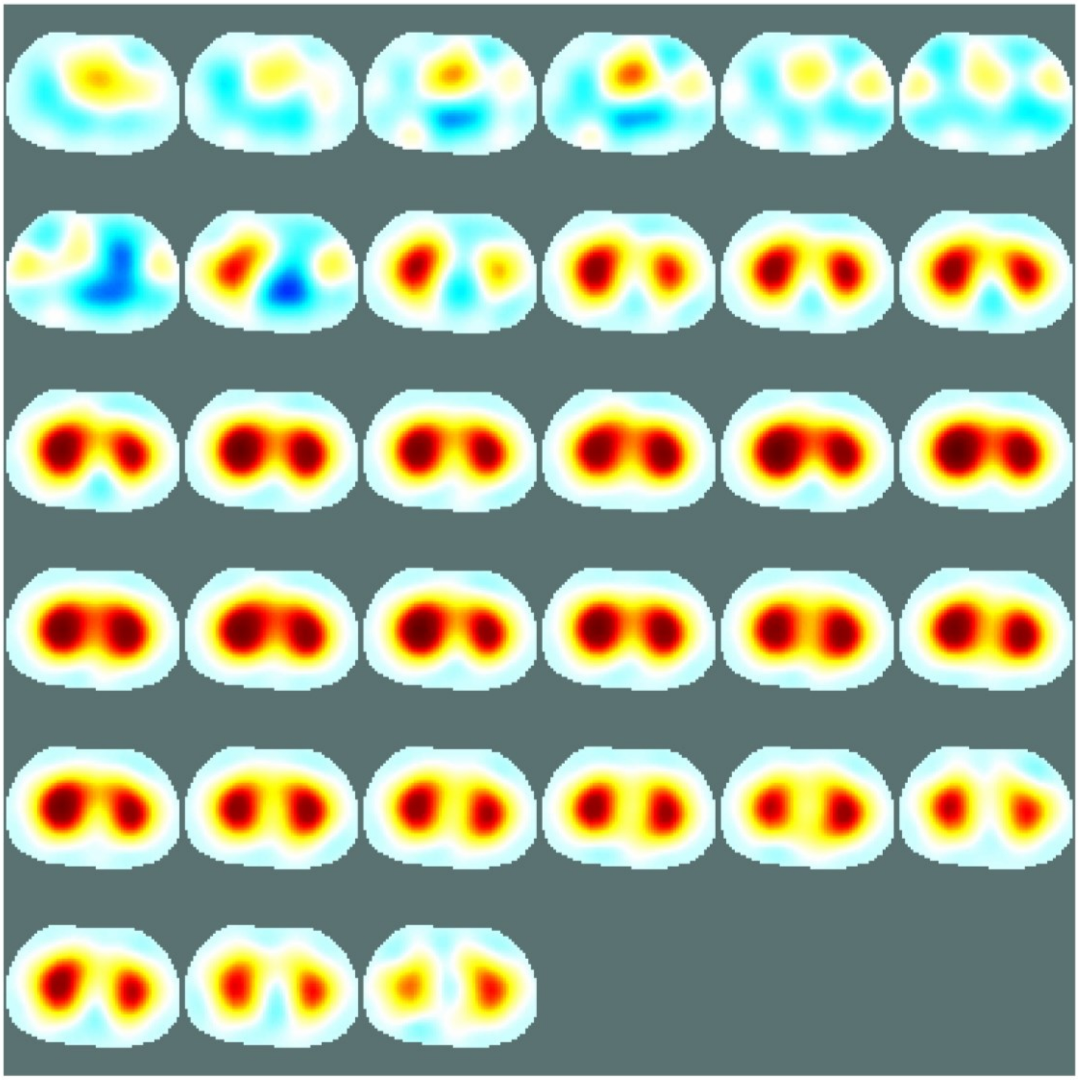

Extended Data Figure 4 33 EIT reconstructions with the iterative PDIPM method, representing a complete breathing cycle from a healthy patient

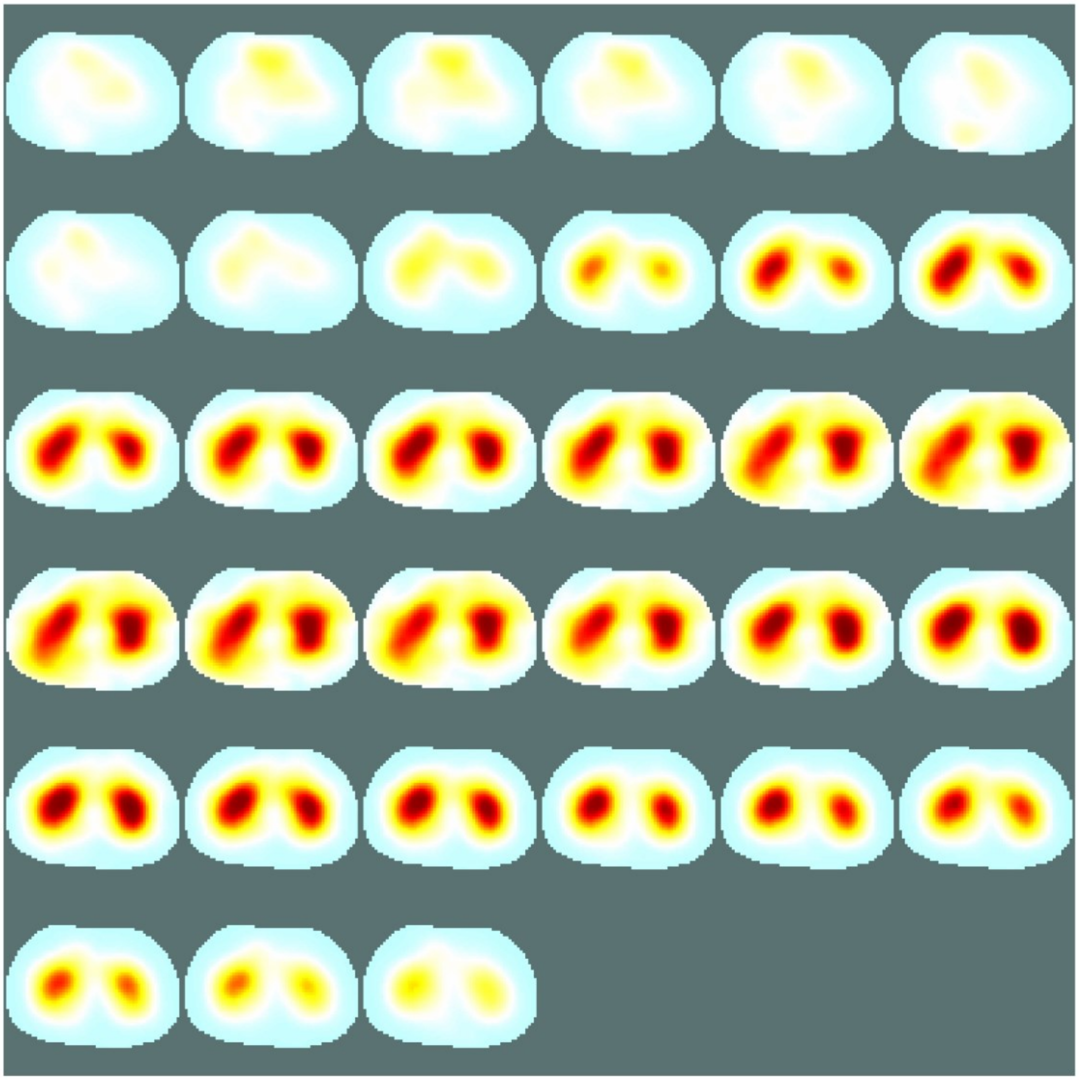

Extended Data Figure 5 33 EIT reconstructions with an ANN used as inverse solver (training includes noise and movement artifacts), representing a complete breathing cycle from a healthy patient

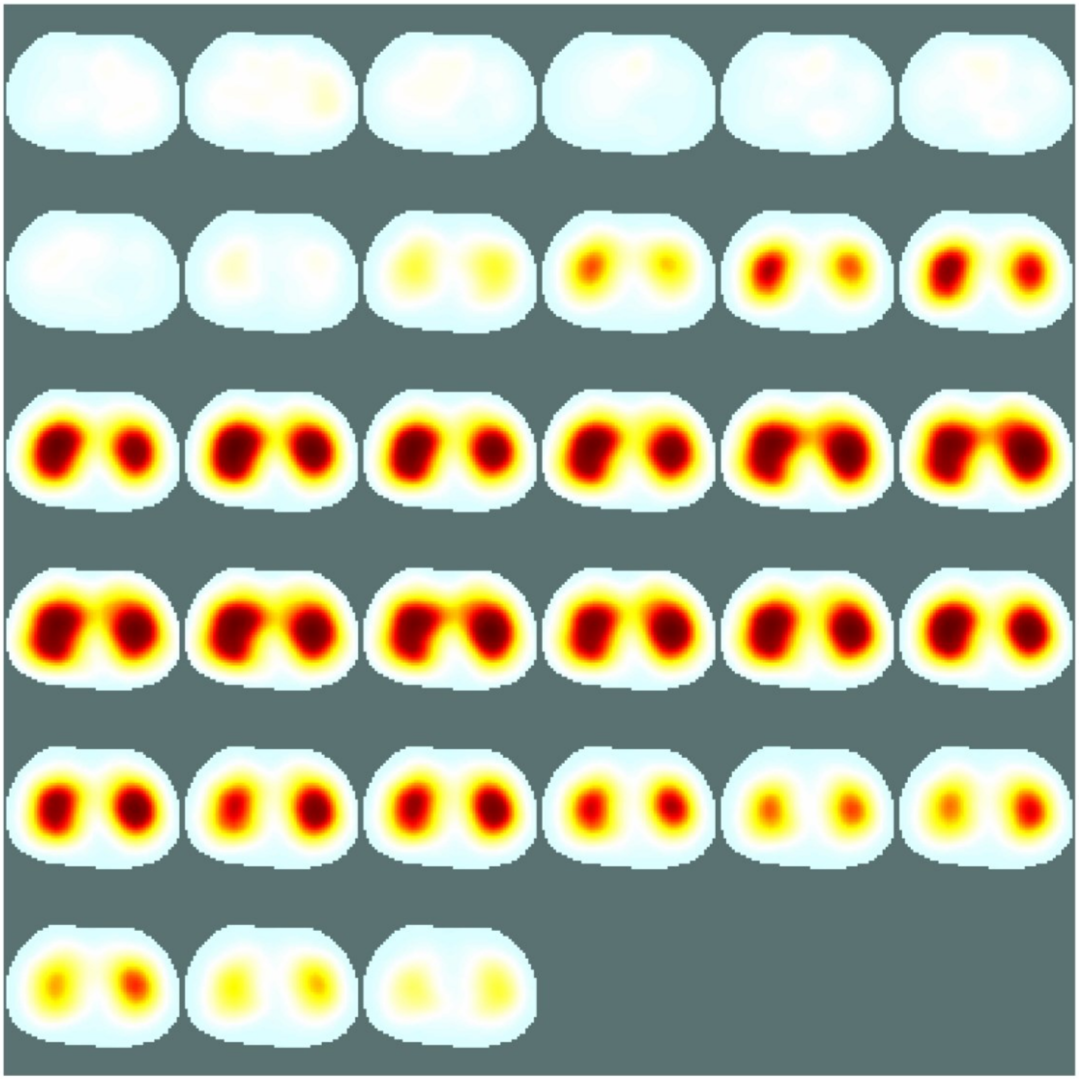

Extended Data Figure 6 33 EIT reconstructions with the proposed post-processing method (training includes noise and movement artifacts), representing a complete breathing cycle from a healthy patient

Extended Data Table 1 |ΔRES| errors measured on two different cross-sections of the 3D model presented in Extended Data Figure 1.

Method	|ΔRES| on one slice taken at 40% of FE model height	|ΔRES| on one slice taken at 60% of FE model height
One-step GN	17.68	18.94
PDIPM	11.01	8.18
ANN (Training: no noise)	8.52	9.62
One-step GN + ANN (training: no noise)	3.95	4.03
ANN (training: noise)	2.93	3.72
One-step GN + ANN (training: noise)	2.74	2.83
